# *Clostridium difficile* Modulates Host Innate Immunity *via* Toxin-Independent and Dependent Mechanism(s)

**DOI:** 10.1371/journal.pone.0069846

**Published:** 2013-07-29

**Authors:** Nazila V. Jafari, Sarah A. Kuehne, Clare E. Bryant, Mamoun Elawad, Brendan W. Wren, Nigel P. Minton, Elaine Allan, Mona Bajaj-Elliott

**Affiliations:** 1 Infectious Diseases and Microbiology Unit, Institute of Child Health, University College London, London, United Kingdom; 2 Clostridia Research Group, Nottingham Digestive Diseases Centre NIHR Biomedical Research Unit, School of Life Sciences, University of Nottingham, Nottingham, United Kingdom; 3 Department of Veterinary Medicine, University of Cambridge, Cambridge, United Kingdom; 4 Gastroenterology Department, Great Ormond Street Hospital, London, United Kingdom; 5 Department of Pathogen Molecular Biology, London School of Hygiene and Tropical Medicine, London, United Kingdom; 6 Research Department of Microbial Diseases, Eastman Dental Institute, University College London, London, United Kingdom; National Jewish Health and University of Colorado School of Medicine, United States of America

## Abstract

*Clostridium difficile* infection (CDI) is the leading cause of hospital and community-acquired antibiotic-associated diarrhoea and currently represents a significant health burden. Although the role and contribution of *C. difficile* toxins to disease pathogenesis is being increasingly understood, at present other facets of *C. difficile*-host interactions, in particular, bacterial-driven effects on host immunity remain less studied. Using an *ex-vivo* model of infection, we report that the human gastrointestinal mucosa elicits a rapid and significant cytokine response to *C. difficile*. Marked increase in IFN-γ with modest increase in IL-22 and IL-17A was noted. Significant increase in IL-8 suggested potential for neutrophil influx while presence of IL-12, IL-23, IL-1β and IL-6 was indicative of a cytokine milieu that may modulate subsequent T cell immunity. Majority of *C. difficile*-driven effects on murine bone-marrow-derived dendritic cell (BMDC) activation were toxin-independent; the toxins were however responsible for BMDC inflammasome activation. In contrast, human monocyte-derived DCs (mDCs) released IL-1β even in the absence of toxins suggesting host-specific mediation. Infected DC-T cell crosstalk revealed the ability of R20291 and 630 WT strains to elicit a differential DC IL-12 family cytokine milieu which culminated in significantly greater Th1 immunity in response to R20291. Interestingly, both strains induced a similar Th17 response. Elicitation of mucosal IFN-γ/IL-17A and Th1/Th17 immunity to *C. difficile* indicates a central role for this dual cytokine axis in establishing antimicrobial immunity to CDI.

## Introduction

In the late 1970s, *Clostridium difficile*, a Gram-positive spore-forming anaerobe was identified as the causative agent of pseudomembranous colitis (PMC), a sporadic but life-threatening gut inflammatory condition [1], [2]. Alarmingly, since 2003 a new lineage of highly virulent *C. difficile* strains has emerged to cause outbreaks of increased disease severity in North America and Europe. Patients infected with these PCR-ribotype 027 strains have more severe diarrhoea, higher mortality and more recurrences [3]. Currently CDI is considered the leading cause of hospital and community-acquired antibiotic-associated diarrhoea in the western world [3]–[5]. This is reflected in the rates of morbidity and mortality with 36,000 cases registered with the UK health protection agency in 2010 alone (www.statistics.gov.uk). CDI is increasingly seen in patient groups which prior to 2000 were considered to be at low-risk, *i.e* those with no recent exposure to antibiotics and in young adults [6]. A higher prevalence of CDI in patients with Inflammatory Bowel Disease (IBD) [7] and Cystic Fibrosis [8] has also been noted.

A better understanding of the microbial pathogenesis of the new virulent strains is a current imperative and significant progress has been made in recent years. Virulent strains can produce up to three toxins: toxin A (TcdA) an enterotoxin, toxin B (TcdB) a cytotoxin and a binary toxin (CDT). *In-vitro* and *in-vivo* studies utilising purified/recombinant TcdA and TcdB, or more recently specific *tcdA/tcdB* insertions have shed light on the probable contribution of each toxin to disease pathogenesis [9], [10]. By contrast, our current understanding of host immunity to *C. difficile* remains rudimentary, particularly as the potent effects of the toxins obscure more subtle host immunity interactions. Our research aims to elucidate *C. difficile*-host interactions that allow elicitation of protective immunity *versus* generation of immunopathology.

In the present study we characterised the human gastrointestinal (GI) mucosal cytokine milieu generated in response to two well described clinical *C. difficile* isolates, R20291 a hypervirulent PCR-ribotype 027 [11] and the fully sequenced strain 630 PCR-ribotype 012 [12]. We found that control colonic tissue had the ability to elicit rapid (as early as 3 h post-infection) and significant cytokine responses to the two strains tested. These observations are important as the mucosal cytokine profile documented is likely to reflect cellular events operative during the acute phase of CDI in humans.

Dendritic cells (DCs) are potent antigen-presenting cells that sense and relay microbial presence to naïve CD4 T cells which, upon activation, differentiate into various effector and regulatory subtypes. In a healthy individual their concerted action culminates in successful microbial clearance while registering immunological memory. Although the effect of purified *C. difficile* TcdA and surface layer proteins (SLPs) on DC function [13]–[15] has been reported, no data is currently available on CDI-mediated DC function and T cell immunity.

Herein, murine bone-marrow-derived and human monocyte-derived DCs (BMDC & mDC) were cultured in the presence of wild-type (WT) strains R20291 and 630. In addition, BMDC responses to *tcdA, tcdB* and *tcdA/tcdB* mutant strains were characterised. The WT strains caused an increase in BMDC maturation marker status and cytokine production; interestingly the majority of parameters measured were not significantly different in response to infection with the toxin-deficient mutants when compared to the WT suggesting that DC activation was mainly bacterial-driven and toxin-independent. The toxin(s) had the greatest impact on IL-1β release, as its secretion was abrogated in response to the double t*cdA/tcdB* knockout (KO) strain. Recombinant TcdA and TcdB elicit potent macrophage IL-1β secretion by activating the inflammasome complex; this process is known to occur *via* Nlrp3 engagement [16]. Interestingly, when we conducted infections in Nlrp3 KO BMDCs, we found minimal reduction in IL-1β levels compared to WT BMDCs; our observations suggests that *C. difficile* has the capacity to engage other, as yet unidentified NLRs during BMDC activation.

Finally, infection with WT strains generated dual Th1/Th17 immunity with R20291 eliciting a significantly more robust Th1 response compared to the 630 strain. Collectively, our study highlights IFN-γ as the central host defence cytokine against *C. difficile*, with IL-22 and IL-17A contributing to the antimicrobial shield. Our data also adds to the growing evidence that the toxin-specific effect on the inflammasome axis may represent a potential therapeutic target for alleviating some of the immunopathology associated with toxigenic *C. difficile* strains.

## Results

### *Ex-vivo* Mucosal Cytokine Responses to *C. Difficile* Strains R20291 and 630

**Figure 1:**
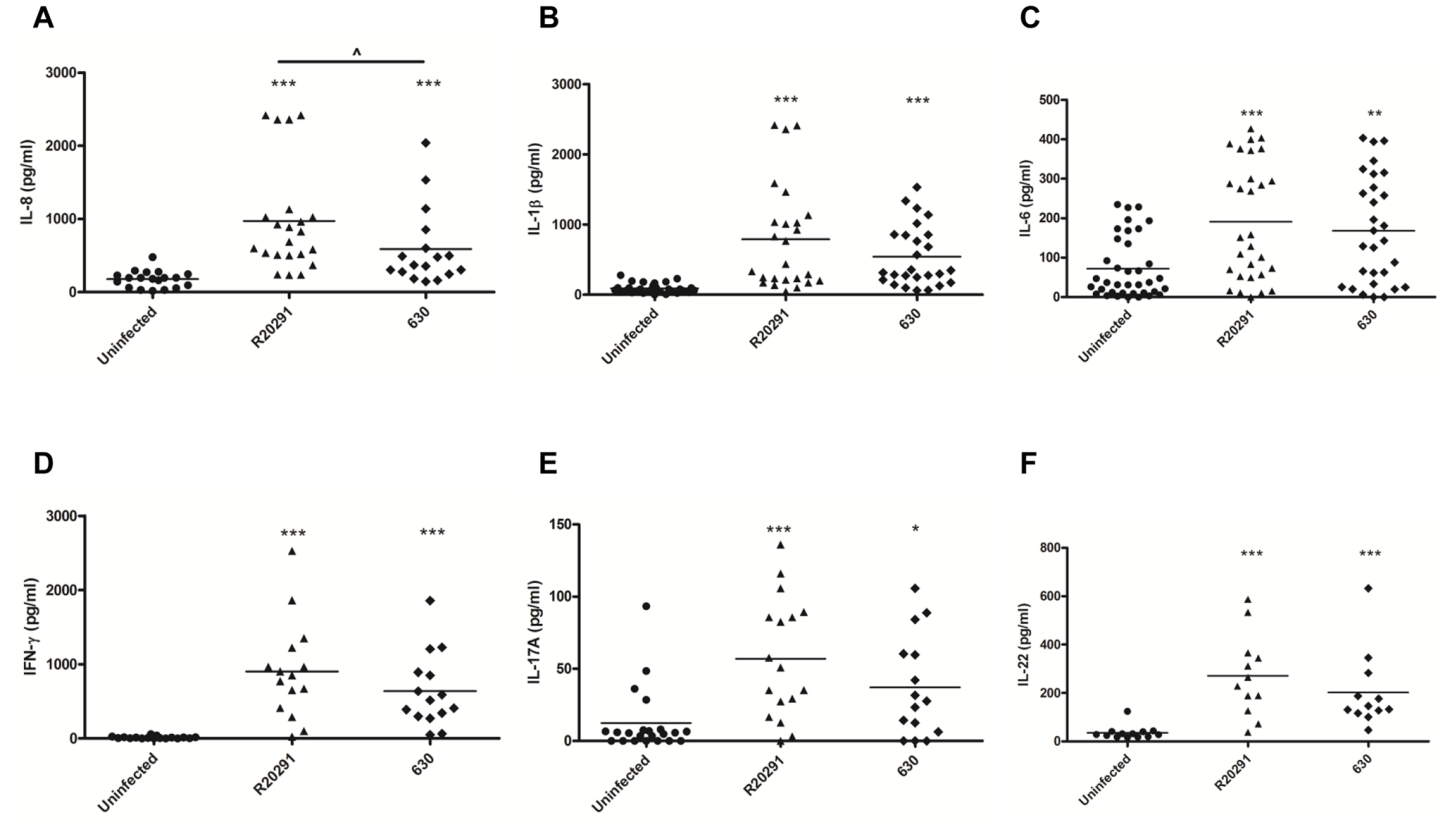
*Ex-vivo* mucosal cytokine responses to *C.* *difficile* infection. Macroscopically normal and matched multiple colonic biopsies (30 individuals with mean age of 10.4± SD of 4.7) were infected with *C. difficile* strains R20291 and 630 (5×10^8^) for 6h. Post-infection IL-8 (A), IL-1β (B), IL-6 (C), IFN-γ (D), IL-17A (E) and IL-22 (F) were quantified by ELISA. Bars represent median levels. *p<0.05, **p<0.01 and ***p<0.001 represent significant difference from uninfected controls and p<0.05 represent significant inter-strain difference. Data was analysed using a Mann–Whitney U test.

The conventional *in-vitro* organ culture (IVOC) method that utilises colonic pinch biopsies has been employed by several investigators to study interaction of enteropathogens with the human gastrointestinal mucosa [17]–[19]. To delineate the mucosal cytokine milieu generated in response to *C. difficile,* colonic biopsy tissue(s) was co-cultured with strains R20291 [hypervirulent ribotype 027 (TcdA^+^/TcdB^+^/CDT^+^) isolated from multi-ward outbreaks in Stoke Mandeville Hospital, UK in 2007] and 630 [ribotype 012 (TcdA^+^/TcdB^+^/CDT^-^) isolated from an outbreak of severe CDI in Zurich in 1985] for 6 h. Upon infection, a marked increase in IL-8 was observed (Fig. 1A). Interestingly, significant inter-strain variation was noted with R20291 mediating greater chemokine release than 630 (p>0.05). IL-1β secretion showed a similar trend (Fig. 1B). IL-6 was detectable in uninfected colonic tissue (Fig. 1C); the increase upon infection was however not as marked as that seen for IL-8 and IL-1β. Infection led to a robust (median 700–850 pg/ml) IFN-γ response (Fig. 1D). The effect on IL-17A expression was modest (Fig. 1E; median 50–60 pg/ml), whilst a significant increase in IL-22 was noted (Fig. 1F; median ∼200 pg/ml).

Overall, the human GI mucosa rapidly responded to *C. difficile* by secreting significant amounts of IL-8, IL-1β and IFN-γ. The consistent greater cytokine response(s) to R20291 suggested that early host-pathogen interactions also render the GI mucosal immune system with the ability to not only distinguish, but also to respond, in a strain-specific manner.

### *C. Difficile*-mediated Effects on Murine bone-marrow-derived Dendritic Cell (BMDC) Activation

**Figure 2:**
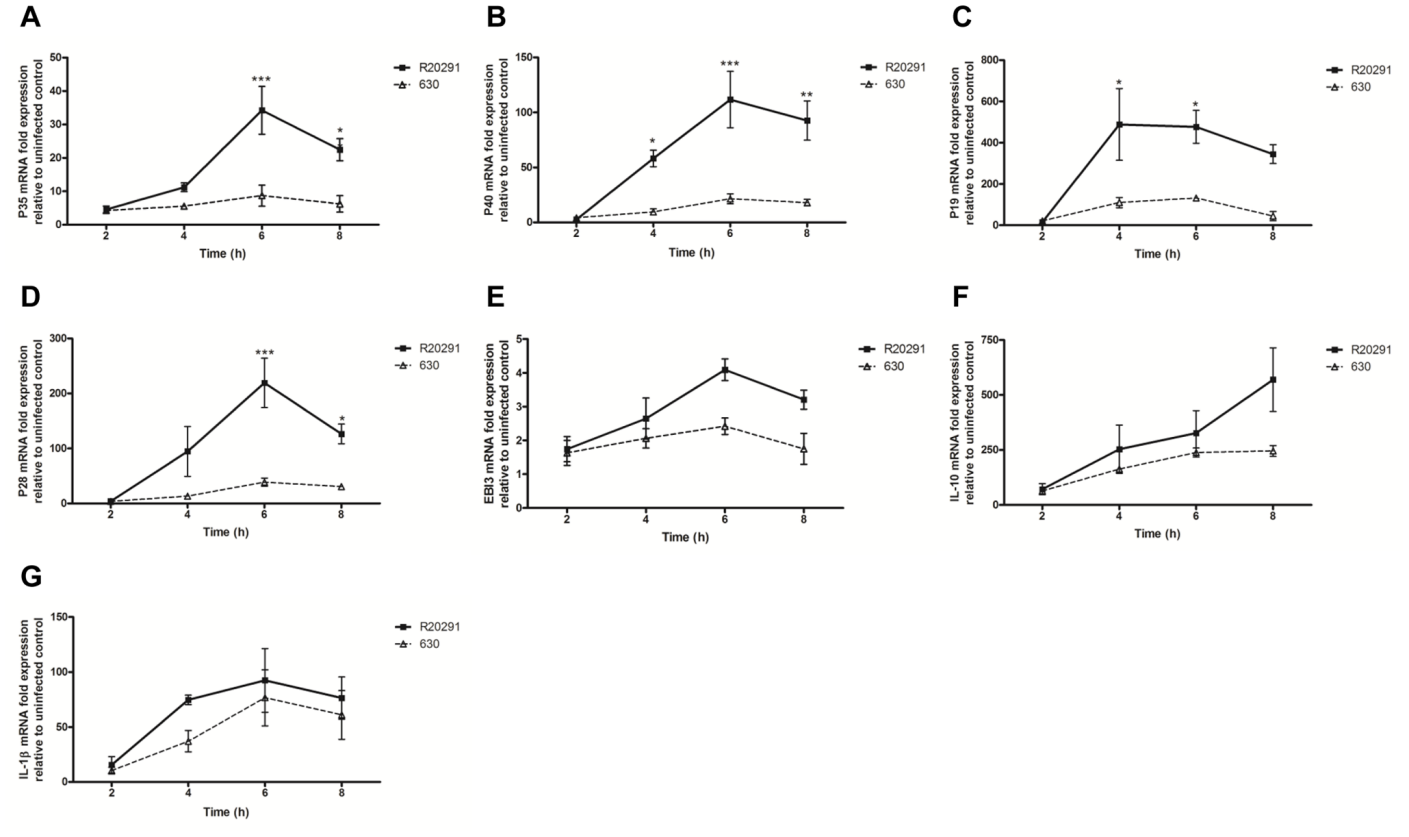
Time-dependent effects of *C.* *difficile* R20291 and 630 infection on BMDC cytokine mRNA expression. BMDCs were infected with bacterial cultures at an MOI of 10 and mRNA expression of IL-12 family members; p35 (A), p40 (B), p19 (C), p28 (D), EBI3 (E), also IL-10 (F) and IL-1β (G) was quantified by real-time PCR. Data is presented as fold increase compared to expression in uninfected control cells. Data represent mean ± SEM, n = 3. *p<0.05, **p<0.001, and ***p<0.001 represent significant inter-strain difference. *P* values were obtained using ANOVA with Bonferroni post-test analysis.

**Figure 3:**
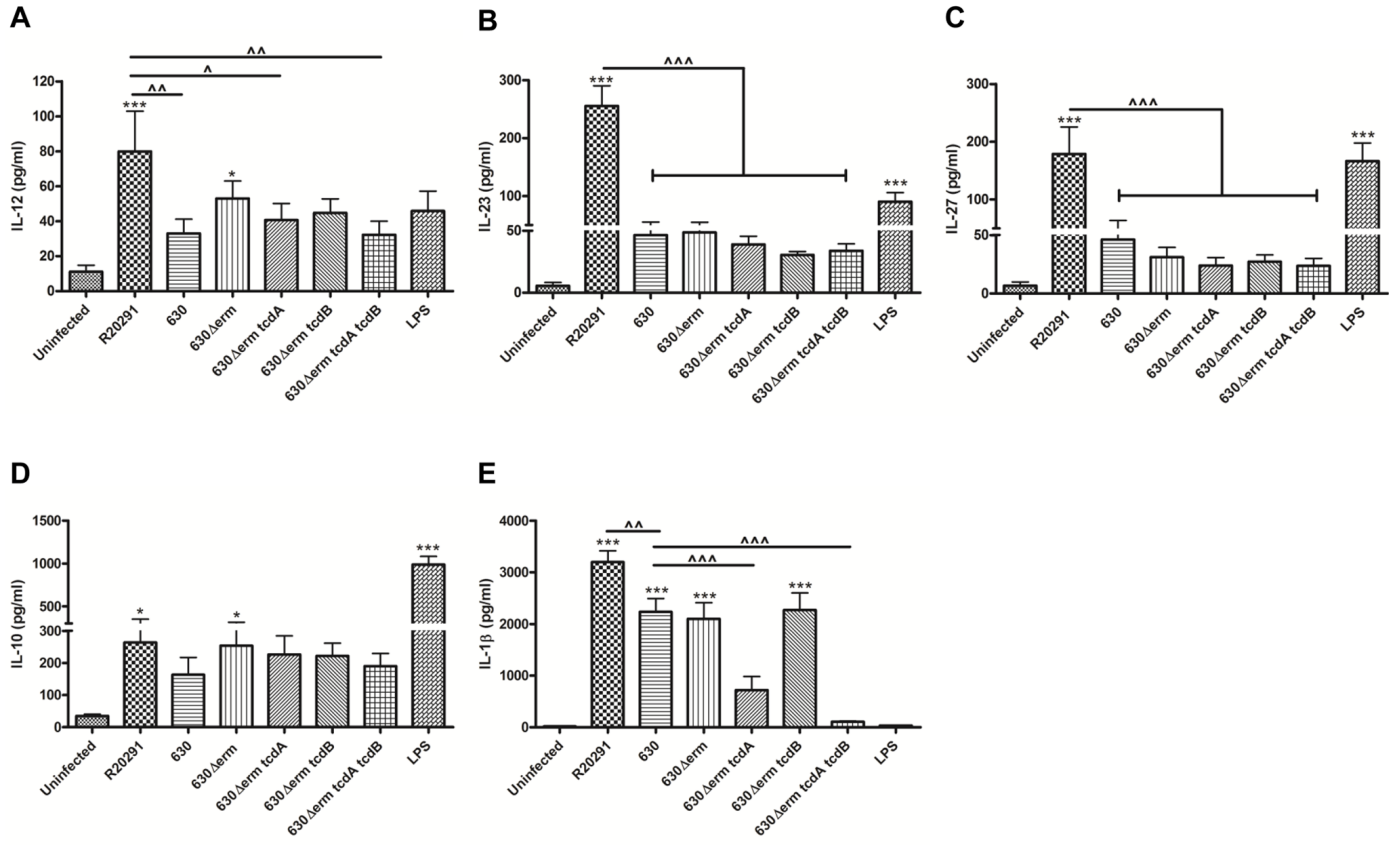
*C.* *difficile*-mediated effects on BMDC cytokine production. BMDCs were infected with *C. difficile* cultures at an MOI of 10 and IL-12 (A), IL-23 (B) IL-27 (C), IL-10 (D) and IL-1β (E) was measured 8 h post-infection. 1 µg/ml LPS stimulation served as positive control. Data represent mean ± SEM of duplicate samples and are representative of three individual experiments. */^∧^p<0.05, ^∧∧^p<0.01 and ***/^∧∧∧^p<0.001 represent significant difference from uninfected cells and significant inter-strain difference. *P* values were obtained using ANOVA with Bonferroni post-test analysis.

To decipher *C. difficile*-mediated DC-T cell crosstalk we employed murine cells as these provide a genetically homogenous model system. Time-dependent effects on BMDC activation in response to WT R20291, WT 630 and 630 toxin isogenic mutant strains were investigated. The intoxication potential of WT 630 and its mutant strains was routinely tested prior to co-culture experiments [10] (Fig. S1A-D in File S1). Modulation of DC maturation status (data not shown) and cytokine response was measured (Fig. 2 & 3). As the toxin mutants were constructed in strain 630Δerm (Table S1 in File S1) this strain was also included in the analysis. Strain 630Δerm is an erythromycin sensitive derivative of strain 630 which was obtained by serial passage [20] and thus may have accumulated mutations compared to 630 [21]. Analysis of the toxin mutants compared to their parent strain enabled us to define bacterial-specific interactions in the absence of the toxins.

### Effect of R20291 and 630 Infections on BMDC Maturation Markers and Cytokine Responses

The capacity of *C. difficile* to modulate BMDC maturation (MHC class II), CD40 and co-stimulatory CD80 and CD86 expression was investigated. Strains R20291, 630 and 630Δerm increased MHC class II and CD40 expression, however this increase did not reach statistical significance and no difference in the magnitude of the responses was apparent with the mutant strains (data not shown). Next, we studied the effect of *C. difficile* on gene and protein expression of candidate cytokines involved in BMDC function (Fig. 2 & 3). IL-12 family members including IL-12, IL-23 and IL-27 are critical determinants of downstream T cell response(s). Heterodimeric IL-12 constitutes subunits p35/p40, IL-23 p19/p40 and IL-27 p28/EBI3. Strains R20291 and 630 both induced p35, p40, p19 and p28 mRNA expression in a time-dependent manner (Fig. 2A–D), with R20291 mediating a statistically significant increase compared to 630, which was most evident 6h post-infection. The increase in EBI3 mRNA did not show a statistical difference between the two strains (Fig. 2E). Interestingly, while eliciting differential responses to the IL-12 family, both strains mediated a similar increase in IL-10 and IL-1β mRNA expression (Fig. 2F & G).

R20291 caused a significant increase not only in IL-12, IL-23 and IL-27 mRNA levels but this was also seen at the protein level (Fig. 3A–C). In comparison, 630 elicited modest levels of all three cytokines. Overall, the absence of toxins did not have a significant impact on the expression of the IL-12 family. Taken together, this data indicates that *C. difficile* strains modulate DC IL-12 members differentially at the transcriptional level; this is an important observation as it suggests that host innate and downstream adaptive immunity may vary depending upon the infecting strain. Unlike the inter-strain variation observed in IL-12/IL-23/IL-27 levels, IL-10, a potent anti-inflammatory cytokine was induced by the WT and 630Δerm strains to a similar extent, and no effect of toxin deletion was noted (Fig. 3D).

We also investigated the effect of *C. difficile* on BMDC IL-β protein levels (Fig. 3E). A marked increase (median ∼2000–3000 pg/ml) in IL-1β secretion was observed in response to the WT strains, with R20291 exerting significantly greater effect compared to 630 and 630Δerm (Fig. 3E). IL-1β secretion was reduced in response to the *tcdA* mutant strain compared to the parental 630Δerm and importantly, was abrogated in response to the *tcdA/tcdB* double-toxin mutant. These observations define the *C. difficile* toxins as central regulators of IL-1β secretion. Overall, our observations suggest that *C. difficile* mediates BMDC activation by engaging toxin-independent mechanism(s) to regulate the IL-12 family and IL-10 responses; in contrast, toxin-dependent mechanism(s) drive IL-1β secretion.

### *C. Difficile* Strains R20291 and 630 Mediate BMDC Inflammasome Activation

**Figure 4:**
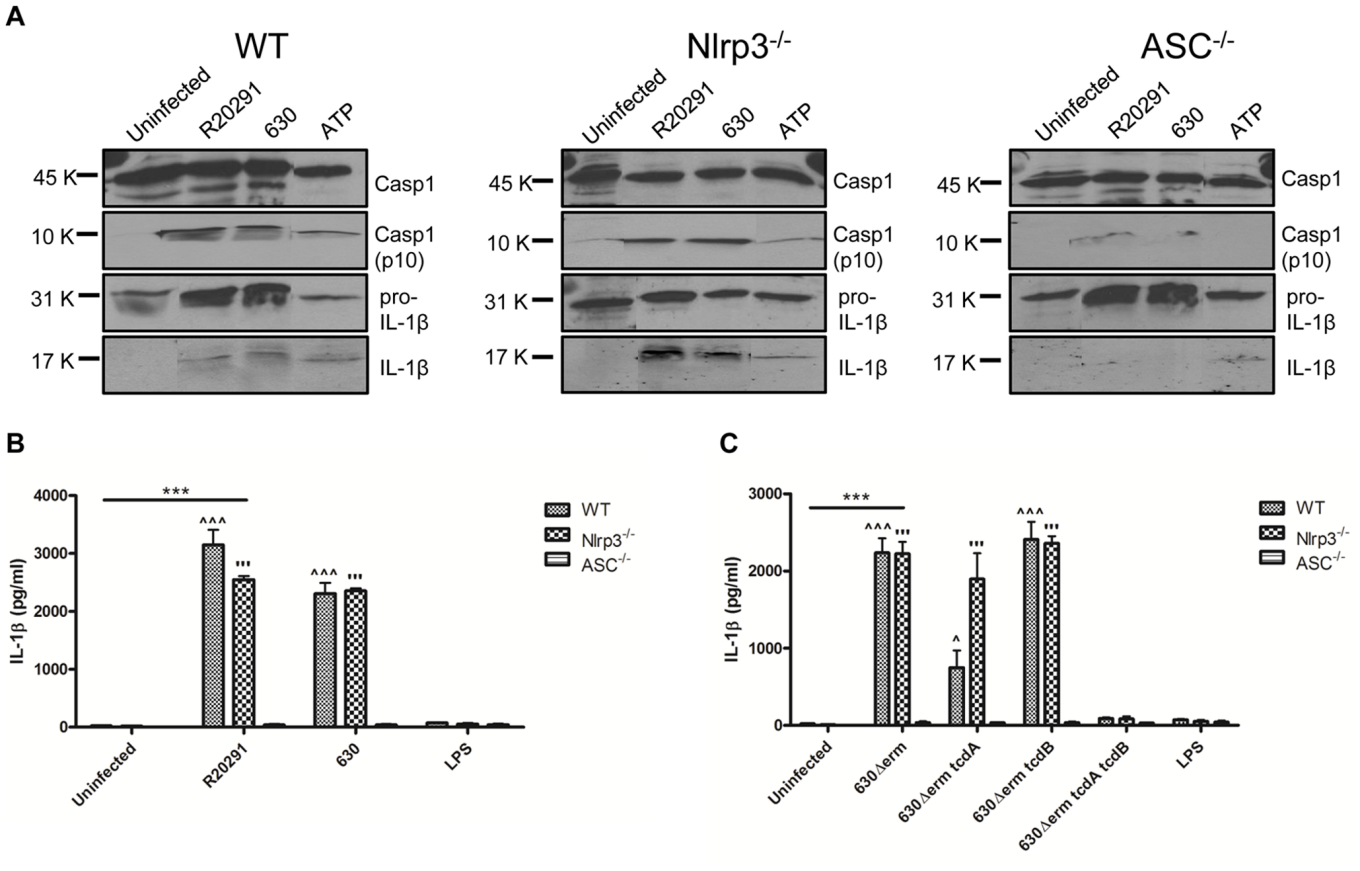
*C.* *difficile* toxins trigger BMDC IL-1β release by activating an ASC-containing inflammasome. BMDCs from WT C57BL/6 (genetically Nlrp1 deficient) mice and mice deficient in Nlrp3 and ASC were infected with *C. difficile* strains at an MOI of 10. Pro- and active caspase-1 (45 & 10 kDa, respectively) and pro- and mature IL-1β (31 & 17 kDa, respectively) release 8h post-infection was quantified by western blotting. 5 mM ATP served as a positive control (A). Quantification of IL-1β secretion in infected WT, Nlrp3 and ASC KO BMDC 8h post-infection. Data is represented as mean ± SEM, n = 4. ***p<0.001 represents significant difference from uninfected cells, ^∧^p<0.05 and ^∧∧∧^/’p<0.001 represent significant WT and Nlrp3^−/−^ difference from ASC^−/−^. *P* values were obtained using ANOVA with Bonferroni post-test analysis (B & C).

Although studies have implicated Nlrp3 in *C. difficile* toxin-mediated inflammasome activation in macrophages [16]; the identity of components involved in BMDC inflammasome function during CDI is unknown. Inflammasome activation culminates in autolytic cleavage of pro-caspase-1 to caspase-1, the enzyme responsible for the cleavage of pro-IL-1β to its bioactive form. Strains were co-cultured with WT, Nlrp3 and ASC KO BMDCs. Cellular caspase-1, pro-IL-1β/IL-1β protein levels and IL-1β secretion were quantified by western blotting (Fig. 4A) and ELISA respectively (Fig. 4B & C). Strains R20291 and 630 showed no major difference in their ability to generate active caspase-1(p10) in WT and Nlrp3 KO BMDCs (Fig. 4A) however, this ability was markedly inhibited in ASC KO BMDCs (Fig. 4A & B), indicating that ASC is a critical component of *C*. *difficile-*mediated BMDC inflammasome activation. In contrast to the 630Δerm *tcdA/tcdB* double-toxin mutant in which IL-1β secretion was absent, infection with the single toxin mutant strains showed no significant difference compared with the parental strain in WT and Nlrp3 KO BMDC (Fig. 4C). Collectively, these experiments suggest that strains R20291 and 630 mediate caspase-1 cleavage in an ASC-dependent manner whilst utilising other upstream NLRs in the absence of Nlrp3.

### R20291 and 630*-*mediated Effects on IL-10 and IL-1β Protein Secretion by Human Monocyte-derived DCs

**Figure 5:**
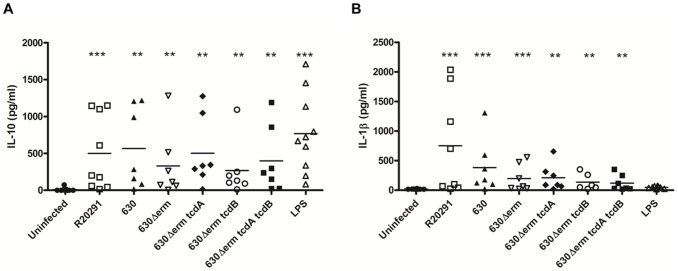
*C.* *difficile-*mediated effects on human monocyte-derived DC IL-10 and IL-1β production. Human mDCs from nine healthy donors were stimulated with *C. difficile* at an MOI of 10. IL-10 (A) and IL-1β (B) secretion measured 8h post-infection. **p<0.01 and ***p<0.001 represent significant difference from uninfected control cells. Data was analysed using Mann-Whitney U test.

As strains R20291 and 630 modulated BMDC cytokine responses in a toxin-independent and dependent fashion (Fig. 2 & 3), we next examined if similar mechanism(s) were also operative during human infection. For this purpose, monocyte-derived DCs (mDCs) from healthy volunteers were infected with the various bacterial strains and IL-10 and IL-1β protein analysed; these two cytokines were chosen as markers of toxin-independent and dependent regulation. The IL-10 protein level in response to WT and mutant strains was similar amongst the donors tested suggesting that *C. difficile* does indeed modulate IL-10 expression in a toxin-independent manner (Fig. 5A). R20291 showed a trend for greater IL-1β secretion compared to 630, and an absence of the single toxin had minimal effect. Interestingly, unlike BMDC IL-1β responses (Fig. 3E) complete abrogation of IL-1β in response to the double-toxin mutant strain was not seen in mDCs (Fig. 5B), indicating that unlike the murine model, toxin-independent bacterial factors may influence inflammasome function in humans.

### R20291 and 630 Infected BMDCs Generate a Cytokine Milieu That Favours Dual Th1/Th17 Immunity

**Figure 6:**
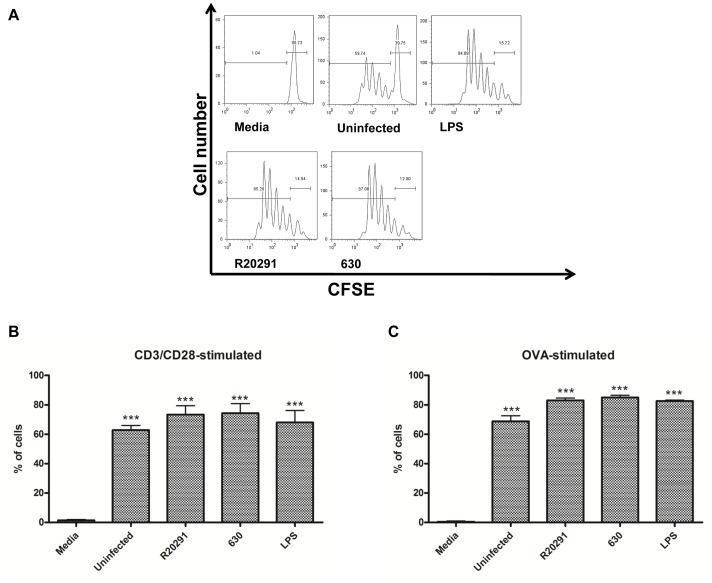
Splenocyte and Naïve CD4^+^ T cell proliferation in response to *C.* *difficile*-stimulated BMDCs. WT BMDCs were infected with PFA-fixed *C. difficile* strains at an MOI of 50. 24 h post-infection, infected BMDCs were co-cultured with CFSE-labelled splenocytes in the presence of anti-CD3/CD28. Proliferation of CD4^+^ gated cells 96 h post stimulation (A). Quantification presented as percentage of proliferating cells (B). Infected BMDCs were co-cultured with CFSE-labelled naïve CD4^+^ T cells from OT-II transgenic mice in the presence of OVA_323–339_ and T cell proliferation was analysed (C). 1 µg/ml LPS utilised as a reference stimulus. ***p<0.001 represent significant difference from uninfected control cells. Data was analysed using ANOVA with Bonferroni post-test.

**Figure 7:**
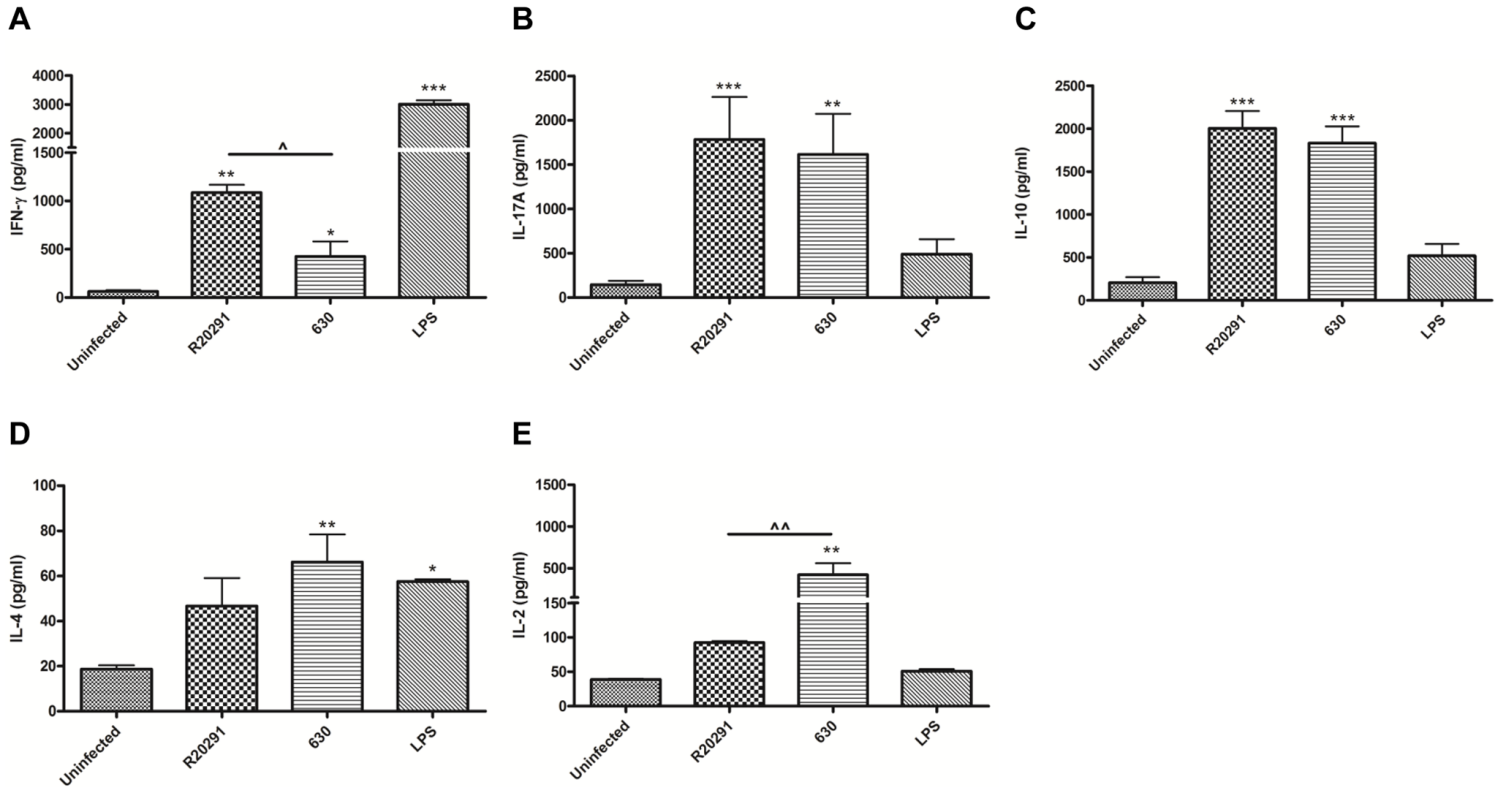
Generation of Th1/Th17 mediated immunity in response to *C.* *difficile* in an *in-vitro* murine model system. WT BMDCs were infected with PFA-fixed *C. difficile* strains. 24 h post-infection, stimulated BMDCs were co-cultured with WT splenocytes in the presence of anti-CD3/CD28. 96 h post co-culture, secreted cytokines IFN-γ (A), IL-17A (B), IL-10 (C), IL-4 (D) and IL-2 (E) were measured by ELISA. 1 µg/ml LPS stimulation served as reference control. Data represent mean ± SEM, n = 3. */^∧^p<0.05, **/^∧∧^p<0.01 and ***p<0.001 represent significant difference when compared to uninfected control cells and significant inter-strain difference, respectively. *P* values were obtained using ANOVA with Bonferroni post-test analysis.

DC-T cell crosstalk is a critical determinant for T cell proliferative capacity and T cell effector function. We investigated T cell proliferation in response to paraformaldehyde (PFA)-fixed R20291 and 630-infected BMDCs in two model systems. Infected BMDCs were co-cultured with CFSE-labelled splenocytes in the presence of anti-CD3/CD28, or with CFSE-labelled naïve CD4 T cells from OT-II transgenic mice in the presence of OVA (peptide _323–339_) [22], [23]. 72–96 h post co-culture, T cell proliferation was quantified by flow-cytometry (Fig. 6A). R20291 and 630-infected BMDCs induced a similar rate of T cell proliferation in both cell systems (Fig. 6B & C). Next, T cell cytokine response(s) were measured (Fig. 7). The number of IFN-γ expressing T cell generated in response to R20291 was significantly greater than seen with strain 630 (Fig. S2A in File S1). This difference was also observed at the protein level (median ∼1000 pg/ml *versus* <450 pg/ml; Fig. 7A). The increase in IL-17A expressing CD4^+^ T cells and IL-17A protein levels was found to be similar in response to both strains (Fig. S2B in File S1 & 7B). In summary, *C. difficile* infection mediated potent Th1 and Th17 immunity and the magnitude of the IFN-γ response was found to be strain-specific. T cell derived IL-10 levels did not significantly differ between the two infectious agents (Fig. 7C). Low IL-4 levels suggested that Th2 immunity plays a minimal role (Fig. 7D). Interestingly, strain 630 showed a greater propensity for IL-2 production compared to R20291 (Fig. 7E).

## Discussion

Despite major scientific breakthroughs in the last century, infectious diseases remain a major global threat to health. The continuous and rapid evolution of bacteria can promote the sudden emergence of strains with increased virulence which poses a new threat to human health. The recent rapid global spread of CDI is one such example. To improve infection control and human wellbeing, greater insight into *C. difficile*-host interactions is warranted. Utilising both murine and human model systems, we present novel findings that significantly extend our understanding of *C. difficile*-mediated crosstalk with the host immune system.

Analysis of the GI mucosal cytokine milieu in the first few hours of *C. difficile* infection revealed robust IL-8 induction (Fig. 1A) with comparatively weak IL-17A response(s) (Fig. 1E) suggesting that the former may play a more crucial role in mediating early neutrophil recruitment, a cellular event increasingly recognised as necessary for *C. difficile* containment. Interestingly, Hasegawa and colleagues [24] reported a minimal role for IL-17A in the acute phase of murine *C. difficile* infection. *C. difficile*-mediated DC-T cell interactions however did lead to marked Th17 immunity (Fig. 7B) emphasising that this cytokine axis may contribute to infection control and/or disease pathogenesis in the chronic phase of infection. Future work must determine if this cytokine family contributes to the dramatic neutrophil influx seen in PMC, a potential serious side-effect of CDI.

Infection with strains R20291 and 630 also mediated significant IL-1β secretion in the *ex-vivo* model of infection (Fig. 1B). To date, *C. difficile* toxins and SLPs have been implicated in mediating IL-1β secretion [13], [16]. To the best of our knowledge the present study is the first to characterise innate immunity in response to *C. difficile* infection. The availability of isogenic mutants lacking individual and both toxins has allowed us to make several novel findings. *Firstly,* elicitation of similar levels of IL-1β in response to 630Δerm and its *tcdB* isogenic mutant strain (Fig. 3E) suggests that expression of a single toxin is sufficient to trigger inflammasome activation, as complete abrogation of function was only noted during infection with the double-toxin mutant strain. Identification of mechanisms responsible for the reduced IL-1β levels in response to strain 630Δerm *tcdA* is currently under investigation. *Secondly*, it is important to note that IL-1β release from human mDCs occurs, albeit at lower levels, even in the absence of toxins (Fig. 5B). This data supports the notion that toxin-independent bacterial determinants may participate in crosstalk with the inflammasome. *Thirdly,* we found redundancy in the role of Nlrp3 function in mediating bioactive IL-β production (Fig. 4B & C) indicating that *C. difficile* has the ability to engage other NLR family members in initiating BMDC inflammasome activation.

A potent mucosal and T cell IFN-γ response was recorded during infection (Fig. 1D & 7A). Our observations place IFN-γ as the central host defence cytokine with IL-22 forming an important component of the early antimicrobial defence (Fig. 1F). Cells responsible for mucosal IFN-γ and IL-22 production require identification.

The differential effect on BMDC IL-12 family with minimal effect on IL-10 and IL-1β expression during infection with the two WT strains was a surprising finding (Fig. 2 & 3). These observations suggest that *C. difficile* has evolved mechanism(s) that allow it to specifically manipulate the IL-12 axis and consequently downstream immunity. Although no significant difference in T cell proliferative responses was noted (Fig. 6), impact of BMDC cytokine milieu on Th1 immunity was apparent. R20291 infection led to a >2-fold increase in the percentage of IFN-γ expressing T cells and cytokine levels (Fig. S2A in File S1 & 7A). In contrast, Th17 immunity was found to be similar (Fig. S2B in File S1 & 7B). Interestingly, strain 630 mediated significantly greater IL-2 responses compared to R20291 (Fig. 7E). IL-2 is a pleiotropic cytokine involved in many aspects of T cell homeostasis including proliferation, differentiation, polarisation and T regulatory function.

In summary, we propose that *C. difficile* strains have evolved to actively modulate DC-T cell crosstalk, an outcome that is likely to be dictated by the genetic content of both the bacterium and the host. In addition, the arsenal of toxins harboured by a particular infecting strain may differentially affect inflammasome activation contributing to the variable severity of *C. difficile* infections seen in humans.

## Materials and Methods

### Ethics Statement

Ethical approval for obtaining mucosal biopsies from patients undergoing routine endoscopic procedure was granted by the Institute of Child Health/Great Ormond Street Hospital (GOSH) Research Ethics Committee (06/Q0508/26). Written informed consent was provided by the legal guardians of the study participants. Blood samples in this study were collected from healthy adult laboratory volunteers approved by the Institute of Child Health Human Tissue Act Review Board. Written informed consent provided by all donors was recorded in accordance with Research Governance and Ethical Regulations of the Institute of Child Health.

C57BL/6 wild-type (WT) mice were purchased from The Jackson Laboratory (Maine, USA) and housed at the Institute of Child Health animal facilities. Approval for animal studies was obtained from University College London Ethics Committee. All experiments were performed according to the United Kingdom Home Office guidelines. NALP3 and ASC deficient murine samples were kindly provided by Dr Panagiotis Tourlomousis (University of Cambridge, UK). Ethical approval was granted by the University of Cambridge Ethics Committee and carried out under UK Home Office project license number 80/2135. OT-II transgenic mice splenic samples were from existing collections kindly given by Dr David Escors (University College London, UK) with the approval of the University College London Ethics Committee.

### Reagents

Brain Heart Infusion (BHI) Agar, BHI broth, *C. difficile* selective supplement and difibrinated horse blood were from Oxoid, Basingstoke, UK. Cysteine, NCTC-135 medium, gentamicin, red blood cell lysing buffer and lipopolysaccharides (LPS) from *Escherichia coli* 0111:B4 were obtained from Sigma-Aldrich, Poole, UK. Dulbecco’s Modified Eagle Medium with GlutaMAX- I (DMEM), Iscove’s Modified Dulbecco’s Medium (IMDM), Roswell Park Memorial Institute (RPMI) 1640, PBS, trypsin-EDTA, 2-Mercaptoethanol and recombinant mouse Granulocyte Macrophage-Colony Stimulating Factor (GM-CSF) were from Invitrogen/Gibco, Paisley, UK. All cell culture media were supplemented with 10% fetal calf serum (FCS), 1% L-glutamine, 1% penicillin-streptomycin solution and 1% non-essential amino acids also obtained from Invitrogen/Gibco.

Anti-Mouse CD80 PE-Cy5, CD86 FITC, MHC Class II FITC, CD40 PE, CD4 PE, IFN-γ PerCP-Cy5.5 and IL-17A FITC were from eBioscience, Hatfield, UK. Antibodies against caspase-1(p10) and IL-1β were acquired from Santa Cruz Biotechnology, inc., USA. Secondary polyclonal rabbit anti-mouse and polyclonal goat anti-rabbit were from Dako, Denmark.

### Bacterial Strains and Growth Conditions

*C. difficile* strains and mutants used in this study are described (Table S1 in File S1). Bacterial strains were cultured on BHI Agar supplemented with 5% difibrinated horse blood or pre-equilibrated BHI broth containing *C. difficile* selective supplement (Oxoid) and 0.05% cysteine. All bacterial cultures were grown in an anaerobic chamber (Don Whitley Scientific, Shipley, UK) in an atmosphere of 10% CO_2_, 10% H_2_, and 80% N_2_ at 37°C.

### Cell Cytoxicity Assay

Human colonic epithelial cell-line HT-29 (ATCC, HTB-38), and African green monkey kidney cells (Vero cells, ATCC, CCL-81) were seeded at a concentration of 1.5×10^5^ and 0.5×10^6^/ml, respectively. Semi-confluent HT-29 and confluent Vero cells were co-cultured with 2-fold serially diluted filter-sterilised bacterial supernatants. The cytopathic effect (CPE) was determined by comparing infected cells to uninfected control cells scoring each dilution on a scale from 0 to +4.

### *In-Vitro* Organ Culture (IVOC)

Pinch biopsies from patients (30 individuals, mean age 10.4± SD of 4.7) were obtained during routine endoscopy. Tissue was oriented with the mucosal surface upwards and mounted on sterile foam supports in 12 well plates. The foams were saturated with IVOC media consisting of complete DMEM media supplemented at ratio of 1∶1 with NCTC-135 medium [25]. The explants were inoculated with 5×10^8^ *C. difficile* at 37°C in 5% CO_2_ humidified incubator for 3–6 h.

### Western Blotting

Bacterial cells and infected-BMDC were washed with ice-cold PBS and lysed in Laemmli buffer (0.5 M Tris-HCl pH 6.8, 10% SDS, glycerol, 0.1% bromphenol blue, 2.5–5% β-mercaptoethanol) and subjected to 15% SDS-PAGE prior to transfer onto a PVDF membrane (Amersham Hybond, GE Healthcare, Little Chalfont, England). Membranes were incubated with appropriate primary antibodies at 4°C overnight prior to exposure to secondary antibodies and detection was by enhanced chemiluminescence (ECL) (Amersham ECL, GE Healthcare).

### Generation and Infection of Murine Bone-marrow-derived DC (BMDC)

Bone-marrow from the femur and tibia of C57BL/6 WT, NALP3/Nlrp3 and ASC knockout (KO) mice was flushed with PBS/2%FCS containing 10 µg/ml gentamicin. The cells were depleted of red blood cells using 1 ml/pair of legs red blood cell lysing buffer. Cells were resuspended in IMDM containing 2, β-mercaptoethanol, gentamycin and GM-CSF. Cells were fed every 2 days. On day 7, cells were harvested by collecting non-adherent cells and detaching adherent cells with 2 mM EDTA-PBS (Ambion, Warrington, UK). Cells were washed and resuspended in complete RPMI without antibiotics and seeded at a density of 1×10^6^/ml prior to infection with *C. difficile* strains at a multiplicity of infection (MOI) of 10. Cytokine protein secretion was measured by ELISA according to the manufacturer’s instructions (eBioscience). Primers for cytokine mRNA detection were from Eurofins MEG Operon, Ebersberg, Germany (Table S2 in File S1) and SYBR Green JumpStart *Taq* ReadyMix was from Sigma-Aldrich. Gene expression of IL-23p19 subunit was analysed using *Taq*Man probe-based PCR from Applied Biosystems.

### Generation and Infection of Human Monocyte-derived DC (mDC)

Blood from healthy donors was collected in the presence of 10 U/ml heparin (CP Pharmaceuticals Ltd, Wrexham, UK). Peripheral blood mononuclear cells (PBMC) were obtained by density gradient centrifugation on Lymphoprep™ (AXIS-SHIELD, Oslo, Norway). CD14^+^ monocytes were separated by the magnetic bead method according to the manufacturer’s instructions (Mini MACS; Miltenyi Biotec GmbH, Germany). Cells were resuspended in complete media (RPMI with 10% FCS, 1% L-glutamine, and 1% penicillin-streptomycin solution) supplemented with 50 ng/ml recombinant human IL-4 and 100 ng/ml recombinant human GM-CSF (R & D Systems, Minneapolis, USA). 0.5×10^6^ cells/ml monocytes were infected with bacterial culture at an MOI of 10.

### T Cell Responses

Splenocytes were harvested from the spleens of WT mice by passing the spleen contents through a cell strainer (70 µm, BD Falcon, BD Biosciences). Cells were washed with PBS and red blood cells depleted using red blood cell lysing buffer. 0.5×10^6^/ml splenocytes (in PBS) were labelled with CFSE [5-(and 6)-Carboxyfluorescein diacetate succinimidyl ester] (eBioscience) to a final concentration of 10 µM. Dynabeads® Mouse T-Activator CD3/CD28 was added to CFSE-labelled splenocytes at a bead-to-cell ratio of 1∶5 then co-cultured with *C. difficile*-stimulated BMDCs in a 96-well plate in a DC: splenocyte ratio of 1∶10. Co-cultures were incubated at 37°C for 72–96 h. Cells were co-stained with anti-mouse CD4 PE-Cy5 (eBioscience) and analysed at 48, 72, and 96 h by flow cytometry gated on CD4^+^ cells.

Naive CD4^+^ T cells were isolated from spleen of OT-II transgenic mice using MagCellect Mouse Naïve CD4^+^ T Cell Isolation Kit (R & D Systems, Inc.) and labelled with CFSE prior to co-culture with *C. difficile*-stimulated BMDCs in the presence of OVA_323–339_ peptide (Abgent, Inc., San Diego, USA) and 10 U/ml recombinant mouse IL-2 (Invitrogen). Cells were co-stained with anti-mouse CD4 PE-Cy5 (eBioscience) and analysed at 48, 72, and 96 h by flow cytometry gated on CD4^+^ T cells.

### Statistical Analysis

Statistical analysis was performed using a two-way ANOVA followed by Bonferonni post-test. A non-parametric *t*-test (Mann–Whitney U-test) was performed on data from *ex-vivo* infections. Data considered significant if only probability was *p*<0.05 using GraphPad Prism version 5.00 (San Diego, CA).

## Supporting Information

File S1 Figure S1, Cytotoxic effects of *C. difficile* toxins on HT-29 and Vero cell-lines. Semi-confluent HT-29 (A & B) and confluent Vero cells (C & D) were co-cultured with 2-fold dilutions of filter sterilised R20291, 630, 630Δerm and its toxin mutant strains. The end-point titre of each dilution series was scored at 8 h post-infection. Data is presented as the mean ± SEM, n = 3. *p<0.05, **p<0.01 and ***p<0.001 represent significant difference from uninfected control cells, ^∧^p<0.05 and ^∧∧^p<0.01 represent significant difference from the parental strain. CD37 is a non-toxigenic strain. *P* values were obtained using ANOVA with Bonferroni post-test analysis. Figure S2, Intracellular IFN-γ and IL-17A staining in CD4^+^ naïve T cells in response to *C. difficile*-stimulated BMDC. Naïve OT-II CD4^+^ T cells were co-cultured with *C. difficile*-stimulated BMDCs in the presence of OVA_323–339_ for 96 h. Intracellular expression of CD4^+^ IFN-γ and IL-17A was analysed by flow cytometry. Data is presented as percentage of IFN-γ^+^ (A) and IL-17A^+^ (B) expressing cells. Data represent mean ± SEM, n = 3. *p<0.05, **^/∧∧^p<0.01 and ***p<0.001 represent significant difference from uninfected control cells and significant inter-strain difference. *P* values were obtained using ANOVA with Bonferroni post-test analysis. Table S1, List of *C. difficile* strains utilised in this study. Table S2, List of primers used in real-time PCR analysis.
(DOCX)
