# Supplementary material for: Clostridium difficile Modulates Host Innate Immunity via Toxin-Independent and Dependent Mechanism(s)
Source: PLoS One. 2013 Jul 29;8(7):e69846. doi: 10.1371/journal.pone.0069846 (PMC3726775; doi:10.1371/journal.pone.0069846)
Supplement: File S1 — Figure S1, Cytotoxic effects of C. difficile toxins on HT-29 and Vero cell-lines. Semi-confluent HT-29 (A & B) and confluent Vero cells (C & D) were co-cultured with 2-fold dilutions of filter sterilised R20291, 630, 630Δerm and its toxin mutant strains. The end-point titre of each dilution series was scored at 8 h post-infection. Data is presented as the mean ± SEM, n = 3. *p<0.05, **p<0.01 and ***p<0.001 represent significant difference from uninfected control cells, ∧p<0.05 and ∧∧p<0.01 represent significant difference from the parental strain. CD37 is a non-toxigenic strain. P values were obtained using ANOVA with Bonferroni post-test analysis. Figure S2, Intracellular IFN-γ and IL-17A staining in CD4+ naïve T cells in response to C. difficile-stimulated BMDC. Naïve OT-II CD4+ T cells were co-cultured with C. difficile-stimulated BMDCs in the presence of OVA323–339 for 96 h. Intracellular expression of CD4+ IFN-γ and IL-17A was analysed by flow cytometry. Data is presented as percentage of IFN-γ+ (A) and IL-17A+ (B) expressing cells. Data represent mean ± SEM, n = 3. *p<0.05, **/∧∧p<0.01 and ***p<0.001 represent significant difference from uninfected control cells and significant inter-strain difference. P values were obtained using ANOVA with Bonferroni post-test analysis. Table S1, List of C. difficile strains utilised in this study. Table S2, List of primers used in real-time PCR analysis. (DOCX) [file pone.0069846.s001.docx]

**FIGURE S1**

**A**

**
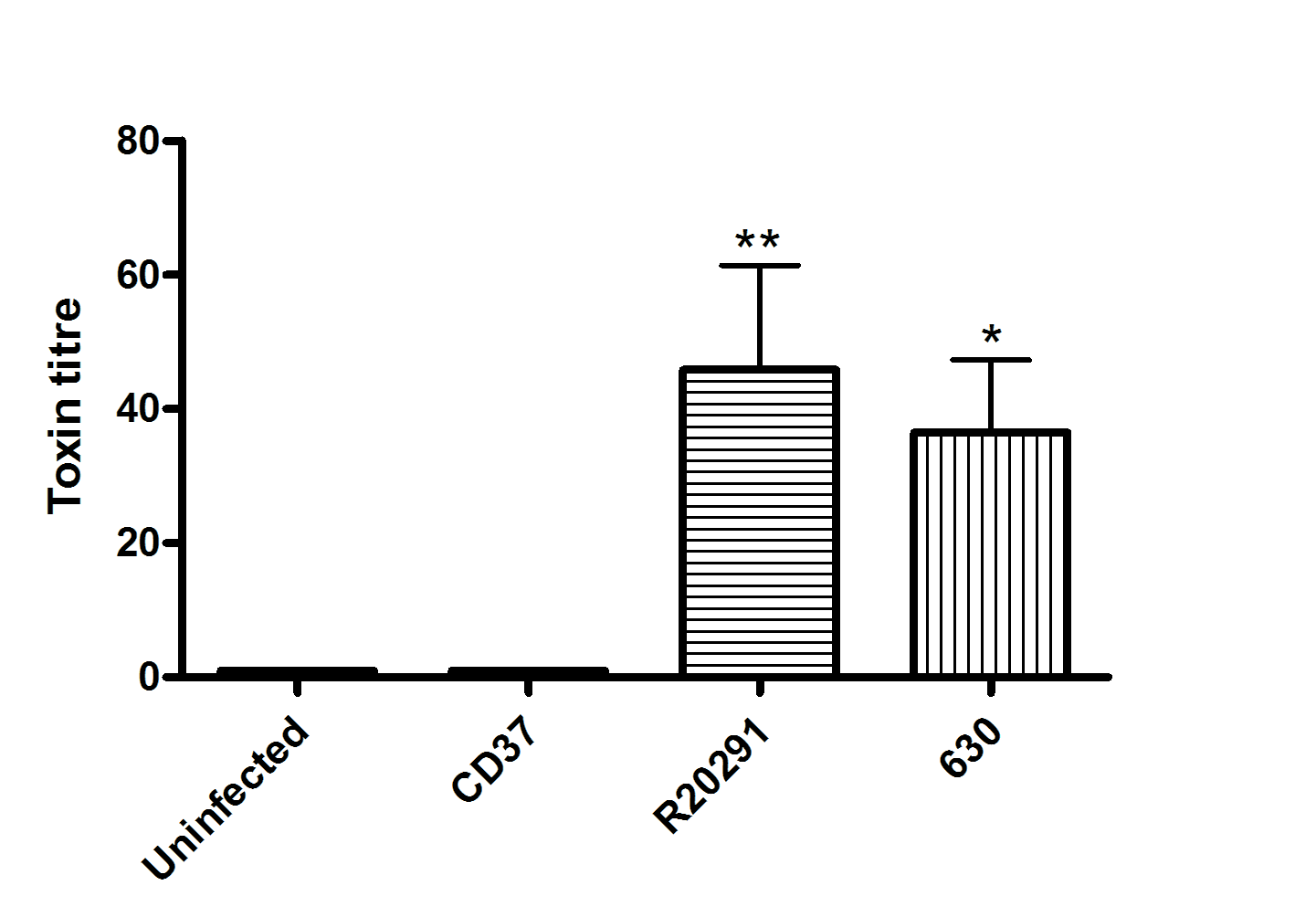
**

**B**

**
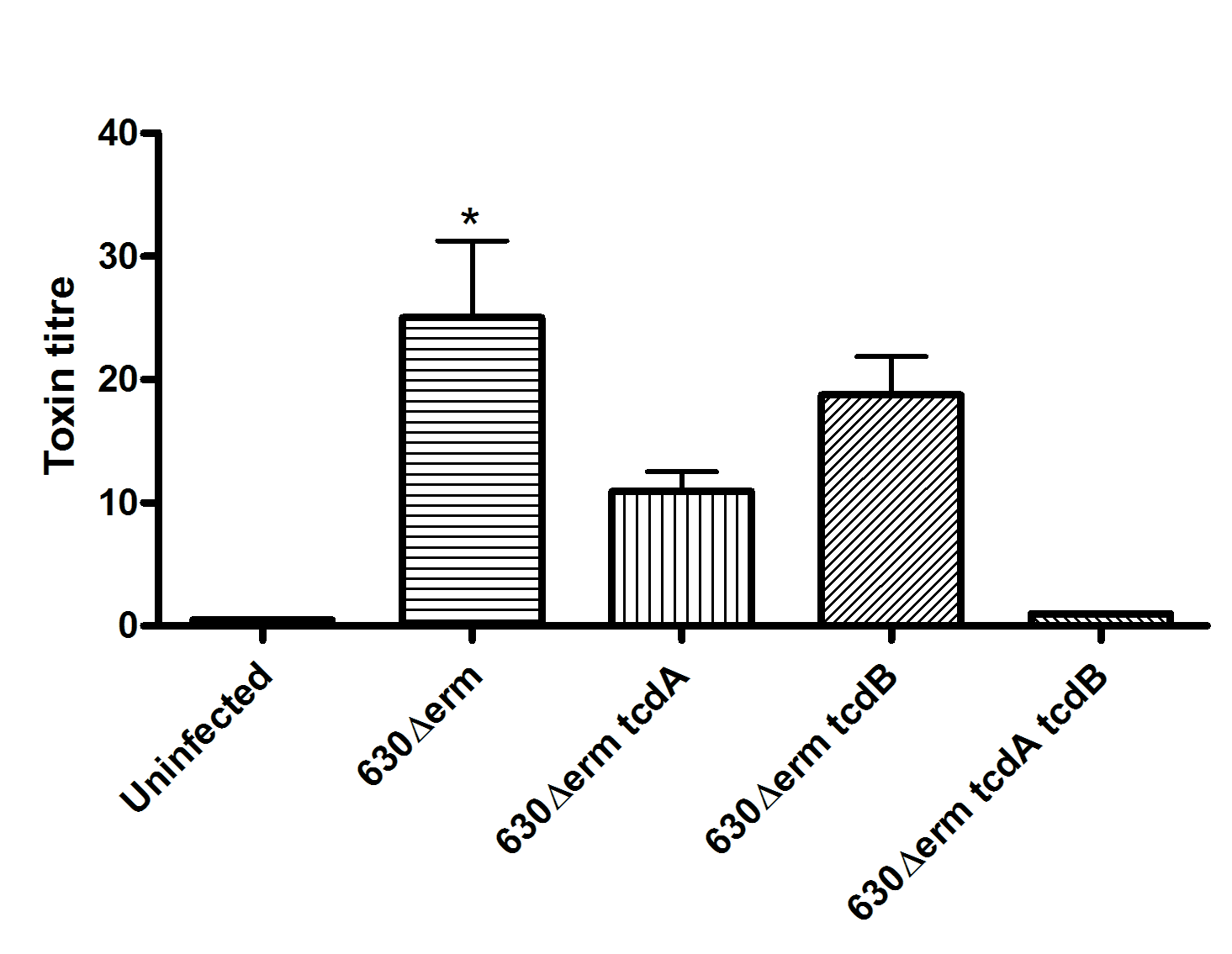
**

**C**

**
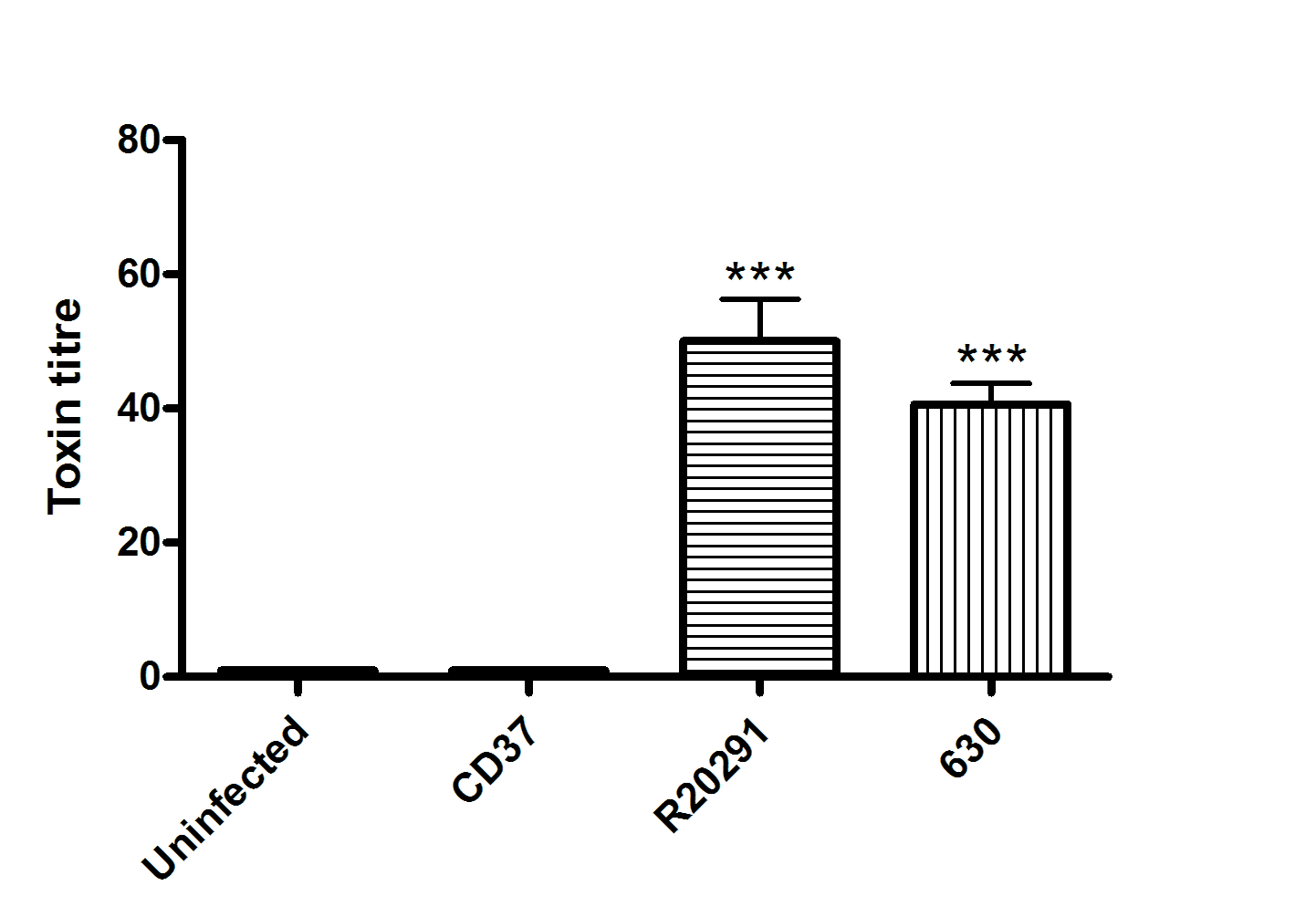
**

**D**

**
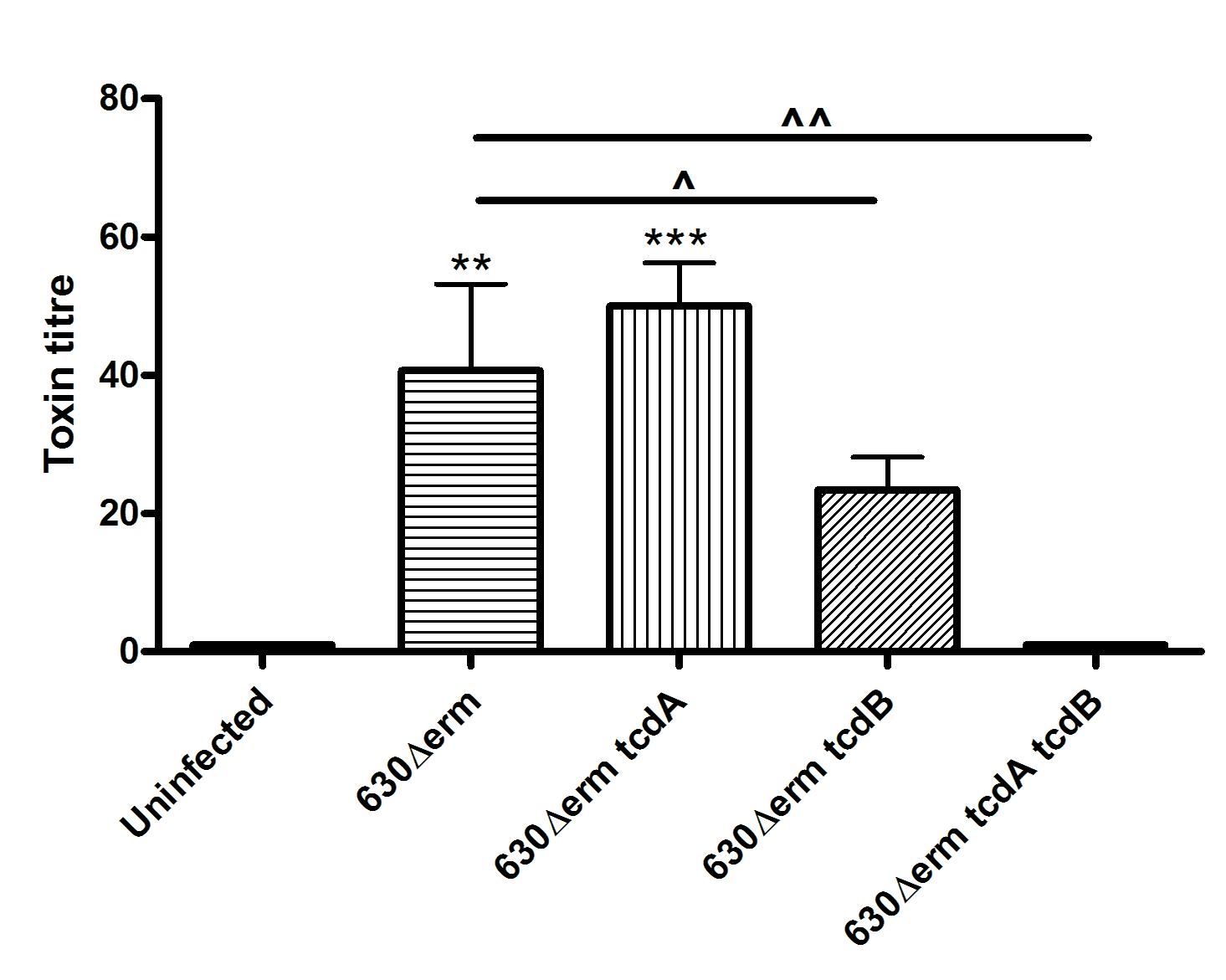
**

**FIGURE S2**

**A**

**
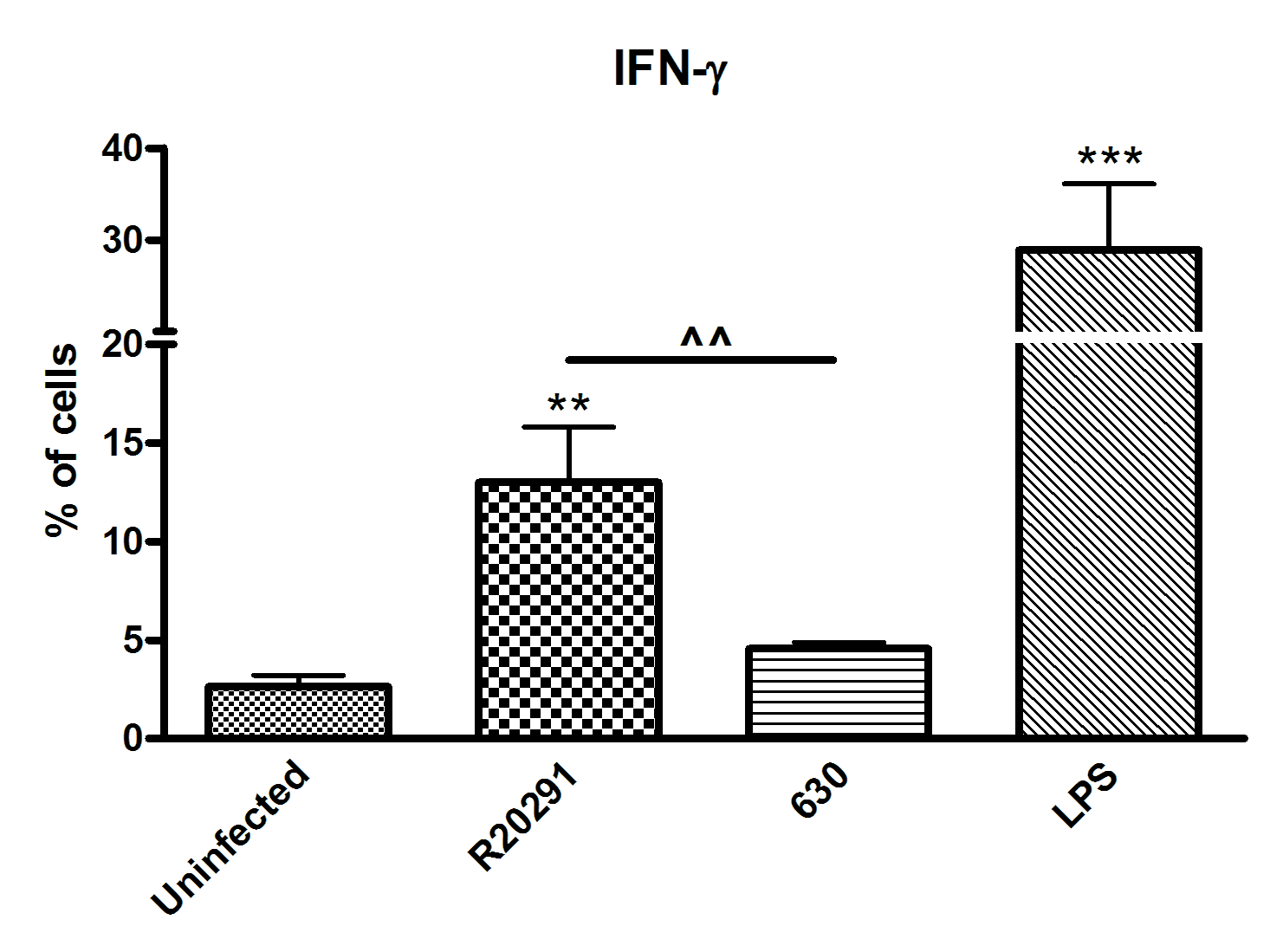
**

**B**

**
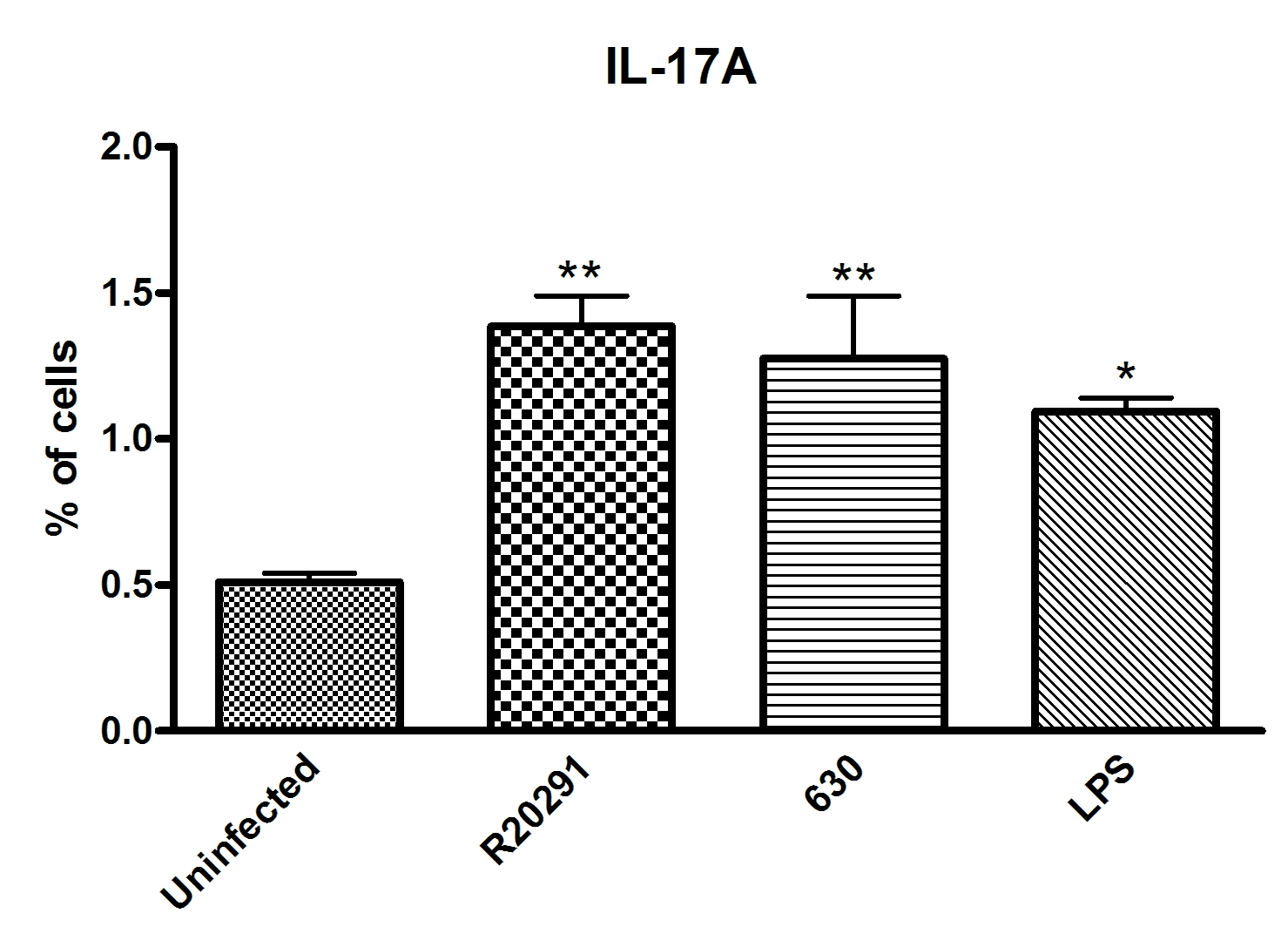
**

**TABLE S1. Bacterial strains**

| **Bacterial strains** | **Produced toxins** | **Ribotype** | **Reference/Source** |
| --- | --- | --- | --- |
| R20291 | A^+^B^+^, CDT^+^ | 027 | P. Mullany, Eastman Dental Institute, UCL, UK |
| 630 | A^+^B^+^ | 012 | [12] |
| 630Δerm | A^+^B^+^ | 012 | [20] |
| 630Δerm *tcdA* | A^-^B^+^ | 012 | [10] |
| 630Δerm *tcdB* | A^+^B^-^ | 012 | [10] |
| 630Δerm *tcdAB* | A^-^B^-^ | 012 | [10] |

**TABLE S2. PCR primers**

|  | **Forward Primer** | **Reverse Primer** |
| --- | --- | --- |
| **IL-12 p35** | 5'-CCTCAGTTTGGCCAGGGTC-3' | 5'-CAGGTTTCGGGACTGGCTAAG-3' |
| **IL12 p40** | 5'-GGAAGCACGGCAGCAGAATA-3' | 5'-AACTTGAGGGAGAAGTAGGAAT-3' |
| **IL-27 p28** | 5′-TTCCCAATGTTTCCCTGACTTT-3’ | 5′-AAGTGTGGTAGCGAGGAAGCA-3’ |
| **IL-27 EBI3** | 5′-TGAAACAGCTCTCGTGGCTCTA-3’ | 5′-GCCACGGGATACCGAGAA-3’ |
| **IL-10** | 5’-GGTTGCCAAGCCTTATCGGA-3’ | 5’-ACCTGCTCCACTGCCTTGCT-3’ |
| **IL-1β** | 5’-CCAAAAGATGAAGGGCTGCT-3’ | 5’-AGAAGGTGCTCATGTCCTCA-3’ |
| **GAPDH** | 5’-CCTGGAGAAACCTGCCAAGTATG-3’ | 5’-AGAGTGGGAGTTGCTGTTGAA-3’ |
